# Predictive Value of Digital Neuropsychological and Gait Assessments on Shunt Outcome in Patients With Idiopathic Normal Pressure Hydrocephalus: Prospective Cohort Study

**DOI:** 10.2196/78399

**Published:** 2025-11-25

**Authors:** Hanlin Cai, Keru Huang, Zilong Hao, Na Hu, Hui Gao, Feng Yang, Shiyu Feng, Linyuan Qin, Ruihan Wang, Xiyue Yang, Shan Wang, Qian Liao, Yi Liu, Dong Zhou, Liangxue Zhou, Jiaojiang He, Qin Chen

**Affiliations:** 1 Department of Neurology, West China Hospital, Sichuan University, No.37 Guoxue Alley, Chengdu, 610041, China, 86 028-85422549; 2 Department of Neurosurgery, West China Hospital, Sichuan University, Chengdu, China; 3 Department of Radiology, West China Hospital, Sichuan University, Chengdu, China

**Keywords:** idiopathic normal pressure hydrocephalus, digital neuropsychological test, gait analysis, external lumbar drainage, lumboperitoneal shunt

## Abstract

**Background:**

The cerebrospinal fluid drainage test is crucial for evaluating patients with idiopathic normal pressure hydrocephalus (iNPH) before shunt surgery, while traditional methods have low sensitivity.

**Objective:**

This study aimed to evaluate the improvement of cognitive and gait parameters after external lumbar drainage (ELD) through the application of digital tests and to investigate the predictive value of digital cognitive and gait assessments for shunt outcomes.

**Methods:**

A total of 70 patients with probable iNPH were enrolled from the West China Hospital of Sichuan University. All patients underwent traditional and digital cognitive and gait assessments at baseline and 3 days after ELD. Thirty-nine patients received lumboperitoneal shunt and were followed up at 3, 6, and 12 months postoperatively using the modified Rankin scale and the Japanese iNPH grading scales. Firth logistic regression models and receiver operating characteristic analysis were used to assess the predictive value of digital tests for shunt response.

**Results:**

The performance of the digital tests, including one-back test (*P*=.01), the Stroop color-word test (*P*=.009), and gait parameters, exhibited significant improvement 3 days post-ELD. Of the 39 shunted patients, 34 exhibited at least 1-point improvement in modified Rankin scale or iNPH grading scales postshunt at their last follow-up. Greater improvement rates in combined digital neuropsychological and gait tests after ELD were associated with a lower risk of unfavorable shunt outcome (adjusted odds ratio=0.98; *P*=.03). Combined digital neuropsychological and gait tests outperformed traditional tests in distinguishing shunt responders (area under receiver operating characteristic curves=0.92 vs 0.55, *P*=.015).

**Conclusions:**

Our study shows that digital neuropsychological and gait tests enhance predictive efficacy when compared to traditional testing methods. It could serve as objective evaluation tools for assessing patients with iNPH.

## Introduction

Idiopathic normal pressure hydrocephalus (iNPH) is a neurological disease characterized by cognitive impairment, gait disturbance, and urinary incontinence [1], with ventricular enlargement and the DESH sign (disproportionately enlarged subarachnoid hydrocephalus) shown on brain scans [2]. It is a common cause of dementia in older people [3] and can be treated effectively with cerebrospinal fluid (CSF) shunts [4,5].

However, the clinical manifestations of iNPH lack specificity, with only 50% of patients exhibiting the full triad [2]. Therefore, patients with iNPH are usually misdiagnosed as other neurodegenerative diseases, such as Alzheimer disease or Parkinson disease [6]. Unlike these conditions, the clinical symptoms of iNPH can be reversed through CSF shunting [7]. However, only 39% to 81% of patients with shunts show improvement 3 to 6 months after the shunt insertion [2]. The CSF drainage test is an important tool for evaluating patients with possible iNPH before shunt surgery [2,8,9]. The tap test and continuous external lumbar drainage (ELD) are 2 commonly used methods [2]. Previous studies have shown that the tap test has a sensitivity of only 26%, whereas the continuous ELD has a sensitivity of 50% and a specificity of 80% [10,11]. As a result, some medical centers conduct continuous ELD directly to avoid repeated lumbar punctures and reduce the risk of misdiagnosis [12-14]. On the other hand, current evaluation methods for CSF drainage tests also have relatively low sensitivities. Previous studies using the Mini-Mental State Examination (MMSE), 10-m walking test (TMWT), and timed up and go (TUG) test indicate a sensitivity of 26% for tap test [10,15]. This implies that current assessment methods are inadequate in providing reliable guidance for clinical practice because traditional cognitive tests can cause large learning effects because of the short time intervals between tests [16] and video-based gait analysis may be influenced by the subjective opinions of raters [17] and lacks quantitative measures, such as stride length, step height, and gait velocity [18,19]. These shortcomings of traditional tests highlight the need for a more objective and quantitative evaluation method.

In recent years, digital neuropsychological evaluation equipment has emerged, which was designed to overcome the major limitations of paper and pencil tests, such as low sensitivity, subjectivity, and test-retest reliability [20]. They also provide randomized test paradigms and automated recording of variables such as reaction time, thereby improving evaluation efficacy [21]. A recent study indicated that computerized neuropsychological tests could detect cognitive impairment and postshunt improvements in patients with iNPH [22]. Additionally, 3-dimensional gait analysis aids in the diagnosis of iNPH by offering multidimensional gait parameters [23,24]. There is growing evidence demonstrating that motor and cognitive impairment in iNPH may result from interconnected neural network impairments [25,26], and recent research based on traditional cognitive and gait tests found that the improvement of different symptoms after ELD may follow different temporal trajectories [27]. Therefore, combining cognitive and gait assessments may provide a more comprehensive and complementary picture of functional response after ELD. Within the expanding field of digital medicine, the integration of digital cognitive and gait tests into diagnostic workflow has been widely investigated and implemented in other cognitive disorders [28,29]. However, an evidence gap remains regarding the predictive value of these digital tests in the context of ELD in patients with iNPH.

Therefore, this study aimed to (1) evaluate the improvement of cognitive and gait parameters after ELD through the application of digital tests and (2) investigate the predictive value of digital cognitive and motor assessments for shunt outcomes in patients with iNPH.

## Methods

### Study Design

Patients with iNPH were enrolled from an ongoing prospective cohort study at West China Hospital of Sichuan University between May 2022 and November 2023. The inclusion criteria included (1) at least one symptom of cognitive decline, gait disturbance, and urinary incontinence; (2) ventricular enlargement (Evans index >0.3), focally dilated sulci, or the DESH sign shown on brain magnetic resonance imaging; (3) CSF opening pressure ≤200 mm H_2_O; and (4) informed consent. We excluded patients with (1) gait disturbance, cognitive impairment, or urinary incontinence due to other neurological diseases (cerebral hemorrhage, brain trauma, brain tumor, and intracranial infection); (2) inability to complete quantitative motor or neuropsychological tests; and (3) refusal to undergo or a negative response to continuous ELD. After enrollment, all participants underwent comprehensive baseline clinical assessments and continuous ELD. Detailed methodologies were described in the following sections. The participant flow throughout the study is summarized in Multimedia Appendix 1. This cohort study was conducted in accordance with the STROBE (Strengthening the Reporting of Observational Studies in Epidemiology) reporting guidelines (Checklist 1).

### Ethical Considerations

This study was approved by the Ethics Committee of the West China Hospital, Sichuan University (No. 2022‐538). To ensure confidentiality, all data were anonymized before analysis and stored securely, with access restricted to the research team. Participants were not compensated financially but volunteered after being informed of the study’s potential benefits.

### Demographic Characteristics

We systematically collected demographic information of the enrolled patients, including age, sex, years of education, medical history, and conducted a comprehensive neurological examination. The Japanese iNPH grading scale (iNPHGS) was used to quantify the severity of symptoms of iNPH [2,30]. Each symptom was scored from 0 to 4, with a total score of 12; higher scores indicating more severe clinical symptoms. The modified Rankin Scale (mRS) was used to assess the patients’ daily living function [7], scored from 0 to 5, with higher scores indicating a more severe inability to perform daily living.

### Neuropsychological Assessments

All neuropsychological tests were conducted by 2 trained neuropsychologists. Traditional neuropsychological assessment uses the MMSE to evaluate global cognitive function [2]. The BrainFit computerized neuropsychological assessment device (Beijing CAS-Ruiyi Information Technology Co, Ltd, China) was used to provide computerized neuropsychological tests, including the grammatical reasoning test, the one-back test, the trail-making test (TMT), and the Stroop color-word test (SCWT). The reliability of the BrainFit system was validated among Chinese populations in previous studies [31,32]. The grammatical reasoning test is designed to evaluate the language comprehension and reasoning ability of the participants [31]. In 60 seconds, they had to determine if the way the shapes were arranged on the screen matched the textual description. The shapes were a circle and a square with a particular positional relationship, and in the middle of the screen was a sentence that described this relationship. If the sentence and shape combination matched, participants clicked “correct”; if not, they clicked “incorrect.” A new question would display within a second of each click [31]. The score for this test was calculated as the number of correct responses minus the number of errors. The one-back test is based on the “N-back” working memory paradigm [33]. Within 60 seconds, participants were required to click the “same” or “different” button to indicate whether they thought the current displayed number matched the previous one. A new number appeared within 1 second after each click. The score was calculated as “the number of correct responses minus the number of incorrect responses” [31]. The Trail Making Test primarily assessed the participant’s attention and reaction speed, requiring the participant to sequentially connect 25 randomly arranged numbers on a page, with the test time recorded [34]. The Stroop Color-Word Test was used to evaluate executive function, psychomotor speed, and cognitive flexibility. During this study, participants were asked to quickly name the color of words that describe different colors, with test time and the number of correct responses recorded [35].

### Gait Analysis

Traditional gait analysis includes the TMWT and the 5-m TUG test [36,37]. The TMWT requires the patient to walk on a 10-m-long path while recording a video. The total time and the number of steps were recorded. The TUG test requires the patient to walk on a 5-m path from one end to the other, turn 180 degrees, and then walk back to the beginning. Each test recorded the overall time, number of steps, and number of turns necessary. Each of these tests was repeated 3 times, and the average of the 3 results was recorded.

Quantitative gait analysis was evaluated using a 3-dimensional gait analysis system ReadyGo (Beijing CAS-Ruiyi Information Technology Co, Ltd), the accuracy and sensitivity of the system have been validated in previous studies [29,38,39]. The ReadyGo system uses a single-camera setup to record 3-dimensional motion by using deep learning for precise positioning of skeletal points. For the single-gait test, participants were instructed to walk at their habitual pace on a 3 m walkway. Step width, stride length, step height, gait velocity, and turning time were the analyzed parameters. Step width was defined as the average width between the left and right feet in each image frame. A single-foot stride length was the distance between 2 landings from the same foot; the overall stride length was the average of the left and right sides. The step height was the height at which a foot could swing without touching the ground. The average step heights of the left and right feet were used for the final analysis. Gait velocity was defined as the distance between the start and end points divided by test time [40].

### Continuous ELD Test

Patients with possible iNPH underwent lumbar puncture and continuous ELD after obtaining informed consent. For 3 to 5 days, the daily CSF drainage volume was controlled between 100 and 150 mL [2,10,13,41]. The patients underwent traditional cognitive and gait assessments at baseline and every day after ELD, with extra digital neuropsychological and gait assessments conducted at baseline and on the third day following ELD. The criteria for a positive response to the traditional evaluation method of ELD included (1) improvement of ≥20% in either time or number of steps in the 10-m walking test or ≥10% improvement in both time and number of steps, (2) improvement of ≥10% in the TUG test time, or (3) improvement of ≥3 points in the MMSE score [2,8,37].

### Postoperative Follow-Up

Patients diagnosed with probable iNPH after ELD underwent lumboperitoneal shunt placement after informed consent was obtained. These patients were followed up regularly at 3 months, 6 months, and 1 year postoperatively. Follow-up was conducted through in-person visits or telephone interviews with the patients and their long-term caregivers. During the in-person visits, comprehensive neurological examinations and brain imaging were performed. Objective assessments of cognitive function, gait, urinary function, daily living abilities, subjective symptom improvements reported by the patients and caregivers, and postoperative complications were documented. Telephone interviews primarily assessed whether the patients and caregivers reported symptom improvement and identified any possible complications. Patients who showed an improvement of ≥1 point in mRS score compared to baseline or an improvement of ≥1 point in any iNPHGS score were defined as definite iNPH (shunt responders) [3,7]. Those who did not report symptom improvements during the follow-up period were classified as nonresponders and underwent pressure readjustment and long-term follow-up.

### Statistical Analyses

The raw scores of grammatical reasoning, the one-back test, TMT, SCWT correct number, and reaction time were converted into standardized Z-scores based on previously reported norms for the Chinese population, facilitating comparisons across tests [34,42]. The z-scores for the Stroop C correct number, grammatical reasoning test, and one-back test were calculated using the formula (raw score−mean)/SD. Z-scores for TMT and Stroop C reaction time were derived by subtracting the raw score from the mean and dividing by the SD. Lower z-scores indicated more significant impairments in the corresponding cognitive domain. The cognitive Z-score for every patient was calculated by taking the mean of the Z-scores from the tests mentioned earlier [36,43]. The improvement rate of the cognitive Z-score was calculated as follows: (post-ELD parameter−pre-ELD parameter)/pre-ELD parameter×100%. For quantitative gait analysis, the improvement rates in gait velocity, stride length, and step height post-ELD were calculated as (post-ELD parameter − pre-ELD parameter)/pre-ELD parameter×100%. The improvement rates of the step width and turning time post-ELD were calculated as follows: (pre-ELD parameter−post-ELD parameter)/pre-ELD parameter×100%. The gait improvement rate was calculated as averages of all 5 gait parameters, and the combined improvement rate was averaged based on the improvement rate of the cognitive Z-score and gait improvement rate [44]. In this pilot study, gait and cognitive improvement were assigned equal weight in the combined improvement rate to reflect global functional improvement after ELD, as the gait and cognitive impairment are core symptoms in iNPH and are considered equally important in current clinical assessments such as iNPHGS [2,30].

For continuous variables, normally distributed data are shown as mean (SD), whereas nonnormally distributed data are presented as median (IQR). Group comparisons were performed using the Student *t* test for normally distributed data and the Mann-Whitney U test for skewed data, whereas comparisons of proportions were performed using the χ^2^ test. Group comparisons before and after the ELD were performed using paired *t* tests for normally distributed data and Wilcoxon signed-rank tests for nonnormally distributed data. To address the limited sample size, we used Firth penalized logistic regression to evaluate the predictive value of improvement rates from each evaluation method for shunt response, and variables demonstrating significant difference in group comparison were included in subsequent multivariate models. This method effectively reduced the small-sample bias and provided more reliable coefficient estimates [45]. The results of Firth logistic regression models were reported by odds ratios (ORs) and receiver operating characteristic curves. Optimal cutoff values were determined based on the Youden index, and diagnostic metrics, including area under the curve (AUC), sensitivity, specificity, positive predictive value, and negative predictive value, were calculated. The DeLong test was used for AUC comparison between traditional tests and digital tests. Permutation tests with 5000 iterations on all Firth regression models and internal validation of the diagnostic metrics with 2000 bootstrap resamples were used to assess the model performance and further enhance the robustness of the statistical inference [46]. A *P* value of .05 was considered statistically significant. All statistical analyses were performed using STATA SE 16.0 (StataCorp, TX, USA) and R version 4.3.0 (R Foundation).

## Results

### Demographic and Clinical Characteristics of Patients With Probable iNPH

**Figure 1.**
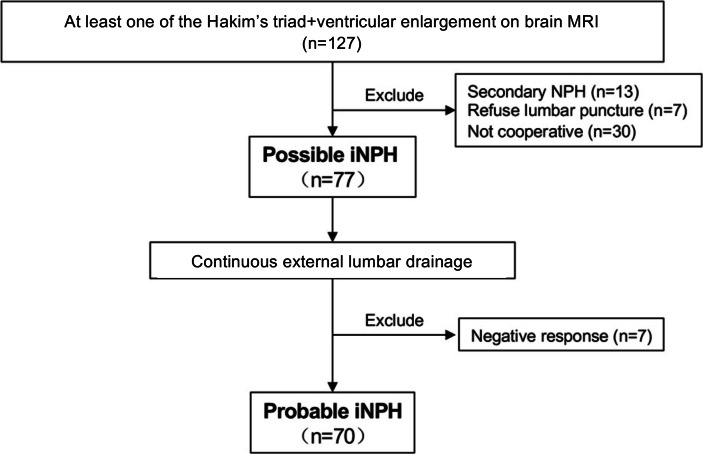
Study flowchart. iNPH: idiopathic normal pressure hydrocephalus; MRI: magnetic resonance imaging.

A total of 127 patients diagnosed with possible NPH were consecutively enrolled in this study (Figure 1). After excluding 7 patients who refused lumbar puncture, 13 patients who developed secondary NPH, and 30 patients who were unable to cooperate with the quantitative motor and cognitive tests, 77 patients with possible iNPH have finally received continuous ELD. Of them, 70 patients were ELD responders and were diagnosed as probable iNPH by clinical neurologists, whereas 7 were negative for ELD tests. Among the 70 patients with probable iNPH, 31 refused shunt surgery and were ultimately diagnosed with probable iNPH, and 39 (55.7%) underwent lumboperitoneal shunt surgery.

Table 1 presents the baseline characteristics of the 70 patients with probable iNPH. The median (IQR) age was 74 (69-80) years, and 70% (49/70) of the patients were male. The prevalence of cognitive impairment, gait disturbances, and urinary incontinence was 92.86% (65/70), 95.71% (67/70), and 54.29% (38/70), respectively, with 51.43% presenting with the full Hakim triad (Multimedia Appendix 2). We compared the baseline characteristics between patients with iNPH who received shunt surgery and those who did not, and the shunted group exhibited a significantly more severe DESH score on brain magnetic resonance imaging (*P*=.007), whereas the 2 groups did not differ in demographics, symptom severity, or improvement rates after ELD. This suggests that the 2 groups were largely comparable at baseline.

**Table 1.** Baseline characteristics of patients with probable iNPH^a^ by the shunt status.

Variables	All (n=70)	No shunt (n=31)	Shunted (n=39)	*P* value
Demographics				
Age^b^ (y), median (IQR)	74.00 (69.00 to 80.00)	74.00 (69.00 to 80.00)	75.00 (71.00 to 78.00)	.64
Sex (male), n (%)	49 (70.00)	19 (61.29)	30 (76.92)	.16
Education level^b^ (y), median (IQR)	12.00 (9.00 to 15.00)	12.00 (9.00 to 12.00)	9.00 (9.00 to 15.00)	.45
Hyperlipidemia, n (%)	28 (40.00)	16 (51.61)	12 (30.77)	.08
Hypertension, n (%)	35 (50.00)	14 (45.16)	21 (53.85)	.47
Diabetes, n (%)	22 (31.43)	8 (25.81)	14 (35.90)	.37
Triad, n (%)	36 (51.43)	12 (38.71)	24 (61.54)	.06
Cognitive decline, n (%)	65 (92.86)	28 (90.32)	37 (94.87)	.46
Gait disturbance, n (%)	67 (95.71)	30 (96.77)	37 (94.87)	.70
Urinary incontinence, n (%)	38 (54.29)	14 (45.16)	24 (61.54)	.17
mRS^b^^c^ (scores), median (IQR)	2.00 (2.00 to 3.00)	2.00 (2.00 to 3.00)	3.00 (2.00 to 3.00)	.34
iNPHGS^d^ (scores), mean (SD)	5.27 (2.22)	4.87 (2.03)	5.59 (2.32)	.18
Evans index^b^, median (IQR)	0.33 (0.31 to 0.35)	0.32 (0.30 to 0.34)	0.33 (0.32 to 0.36)	.10
DESH^b^^e^ score, median (IQR)	6.00 (5.00 to 7.00)	5.00 (4.00 to 7.00)	6.00 (6.00 to 7.00)	.007^**f**^
Baseline neuropsychological tests				
MMSE^b^^g^(score), median (IQR)	21.00 (14.00 to 25.00)	19.00 (13.00 to 24.00)	21.00 (15.00 to 25.00)	.65
Grammatical reasoning^b^ (score), median (IQR)	1.00 (0.00 to 3.00)	1.00 (0.00 to 2.00)	1.00 (0.00 to 3.00)	.53
One-back test^b^ (score), median (IQR)	5.00 (2.00 to 9.00)	6.00 (2.00 to 9.00)	5.00 (2.00 to 9.00)	.95
Trail-making test^b^ (s), median (IQR)	140.00 (103.00 to 150.00)	140.00 (103.00 to 150.00)	140.00 (122.00 to 150.00)	.98
Stroop color-word test^b^ (score), median (IQR)	36.00 (14.00 to 45.00)	37.00 (34.00 to 46.00)	34.00 (14.00 to 42.00)	.28
SCWT^b^^h^ reaction time (s), median (IQR)	150.00 (116.56 to 186.65)	154.56 (142.25 to 222.46)	148.92 (106.41 to 183.66)	.10
Cognitive z-score^d^, mean (SD)	−2.35 (1.19)	−2.46 (1.27)	−2.26 (1.12)	.51
Baseline gait tests				
5-m^b^^i^ TUG (s), median (IQR)	18.00 (15.00 to 27.00)	22.00 (16.00 to 30.00)	17.00 (15.00 to 22.00)	.13
TMWT^b^^j^ (s), median (IQR)	15.00 (12.00 to 22.00)	17.00 (13.00 to 25.00)	15.00 (12.00 to 18.00)	.13
TMWT steps^b^, median (IQR)	26.00 (21.00 to 38.00)	30.00 (23.00 to 42.00)	25.00 (19.00 to 33.00)	.06
Step width^b^ (m), median (IQR)	0.16 (0.14 to 0.17)	0.15 (0.14 to 0.17)	0.16 (0.15 to 0.16)	.68
Stride length^d^ (m), mean (SD)	1.30 (0.50)	1.28 (0.49)	1.31 (0.50)	.81
Step height^d^ (m), mean (SD)	0.08 (0.03)	0.08 (0.02)	0.08 (0.03)	.38
Gait velocity^d^ (m/s), mean (SD)	0.68 (0.22)	0.66 (0.25)	0.69 (0.20)	.70
Turning time^b^ (s), median (IQR)	2.22 (1.57 to 3.40)	2.22 (1.57 to 2.95)	2.28 (1.73 to 3.57)	.47
Improvement rate after ELD^k^				
Cognitive improvement^b^ (%), median (IQR)	19.5 (−15.9 to 41.9)	27.9 (−5.3 to 47.6)	2.0 (−17.3 to 32.1)	.14
Gait improvement^b^ (%), median (IQR)	5.1 (−3.5 to 15.1)	5.5 (−3.5 to 12.6)	5.1 (−0.4 to 14.8)	.99
Combined improvement^b^ (%), median (IQR)	12.4 (−6.5 to 26.7)	16.7 (5.8 to 30.1)	3.2 (−7.9 to 21.6)	.12

a iNPHGS: iNPH grading scale.

b The Mann-Whitney *U* test was used for group comparisons.

c mRS: modified Rankin scale.

d The Student *t* test was used for group comparisons.

e DESH: disproportionately enlarged subarachnoid hydrocephalus.

f Statistically significant.

g MMSE: Mini-Mental State Examination.

h SCWT: Stroop color-word test.

i TUG: timed up and go test.

j TMWT: 10-m walking test.

k ELD: external lumbar drainage.

**Figure 2.**
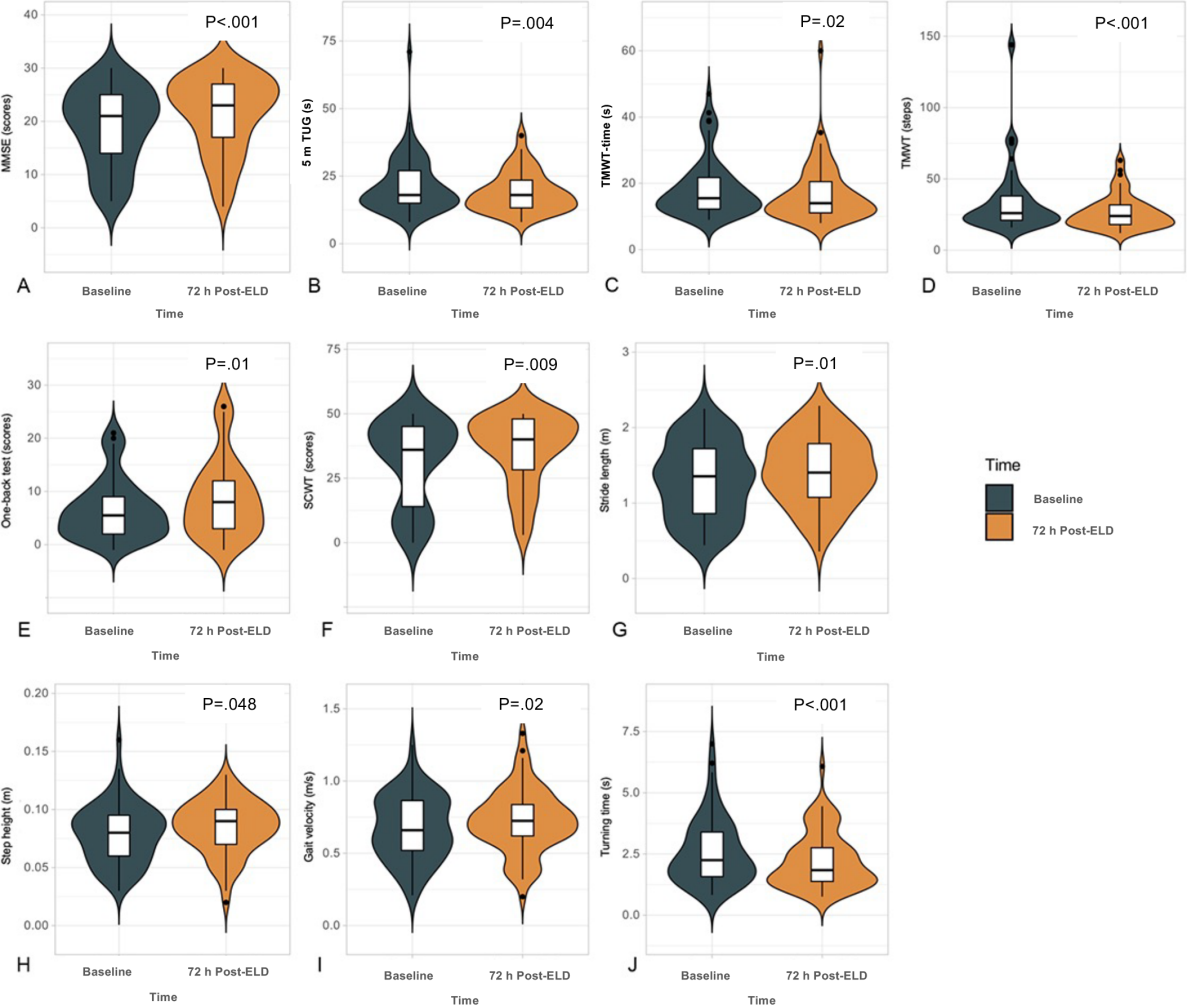
Changes in cognitive and gait parameters after 3-d ELD. (A) Mini-Mental Status Examination (MMSE); (B) 5-m timed up and go test (5m-TUG); (C) 10-m walking test (TMWT) time; (D) 10-m walking test (TMWT); (E) one-back test; (F) stroop color-word test (SCWT); (G) stride length; (H) step height, (I) gait velocity, and (J) turning time. ELD: external lumbar drainage.

As shown in Figure 2 and in Multimedia Appendix 3, among the 70 patients with probable iNPH, traditional tests, including MMSE (*P*<.001), TUG (*P*=.004), TMWT time (*P*=.016), and steps (*P*<.001), showed significant improvement 3 days after ELD. For digital tests, one-back test (*P*=.01), SCWT correct numbers (*P*=.009), and composite cognitive z-scores (*P*=.025) improved significantly 3 days after ELD. Quantitative gait analyses also showed significant improvement in stride length (*P*=.01), step height (*P*=.048), gait velocity (*P*=.019), and reduced turning time (*P*<.001) after 3-day ELD compared to baseline.

### Predictive Value of Digital Neuropsychological and Gait Tests for Shunt Outcome in Patients With iNPH

A total of 39 patients with probable iNPH underwent lumboperitoneal shunt placement; after a median follow-up interval of 116 days, 34 (89.7%) showed at least a 1-point improvement in mRS or iNPHGS, leading to a clinical diagnosis of definite iNPH or being classified as “shunt responders,” 4 patients only reported subjective symptomatic improvement but not measurable through mRS or iNPHGS, and 1 patient did not report any improvement at the time of final follow-up and was managed with pressure readjustment and continued follow-up.

First, we compared the clinical characteristics between shunt responders and nonresponders (Table 2). The results demonstrated a higher proportion of male sex (*P*=.036), lower Evan index (*P*=.035), and significantly higher improvement rate of gait after ELD (*P*=.04) for shunt responders, whereas they did not differ significantly in age, educational level, or vascular risk factors. Multivariate Firth logistic regression model adjusted for sex and Evan index showed a significant association between a higher improvement rate of digital gait analysis and a lower risk of unfavorable shunt outcome (adjusted OR 0.90, 95% CI 0.78‐0.99; *P*=.03). In addition, an association between a higher combined improvement rate of digital neuropsychological and gait analysis and a lower risk of unfavorable shunt outcome was also observed (adjusted OR 0.98, 95% CI 0.95‐1.00; *P*=.03; Multimedia Appendix 4).

**Table 2.** Clinical characteristics of iNPH^a^ patients who received lumboperitoneal shunt.

Variables	All (n=39)	Subjective improvement and nonresponders (n=5)	Objective responders (n=34)	*P* value
Age (y), median (IQR)	75.00 (71.00 to 78.00)	67.00 (66.00 to 73.00)	76.00 (71.00 to 81.00)	.10
Sex, male, n (%)	30 (76.92)	2 (40.00)	28 (82.35)	.04^b^
Education level (y), median (IQR)	9.00 (9.00 to 15.00)	9.00 (9.00 to 12.00)	9.00 (9.00 to 15.00)	.49
Hyperlipidemia, n (%)	12 (30.77)	2 (40.00)	10 (29.41)	.63
Hypertension, n (%)	21 (53.85)	2 (40.00)	19 (55.88)	.51
Diabetes, n (%)	14 (35.90)	3 (60.00)	11 (32.35)	.23
Evans index, median (IQR)	0.33 (0.32 to 0.36)	0.38 (0.34 to 0.38)	0.33 (0.31 to 0.34)	.04^b^
DESH^c^ score, mean (SD)	6.31 (1.32)	5.80 (0.98)	6.38 (1.35)	.37
Cognitive improvement rate after ELD^d^, %, median (IQR)	2.01 (−17.35 to 32.07)	−10.56 (−31.43 to 28.94)	2.01 (−17.35 to 33.55)	.47
Gait improvement rate after ELD, %, median (IQR)	5.07 (−0.38 to 14.77)	−12.32 (−14.83 to −2.27)	5.24 (1.63 to 17.05)	.04^b^
Combined improvement rate after ELD, %, median (IQR)	3.17 (−7.93 to 21.58)	−12.70 (−16.85 to 14.34)	3.17 (−6.47 to 26.72)	.28

a iNPH: idiopathic normal pressure hydrocephalus.

b Statistically significant.

c DESH: disproportionately enlarged subarachnoid hydrocephalus.

d ELD: external lumbar drainage.

**Figure 3.**
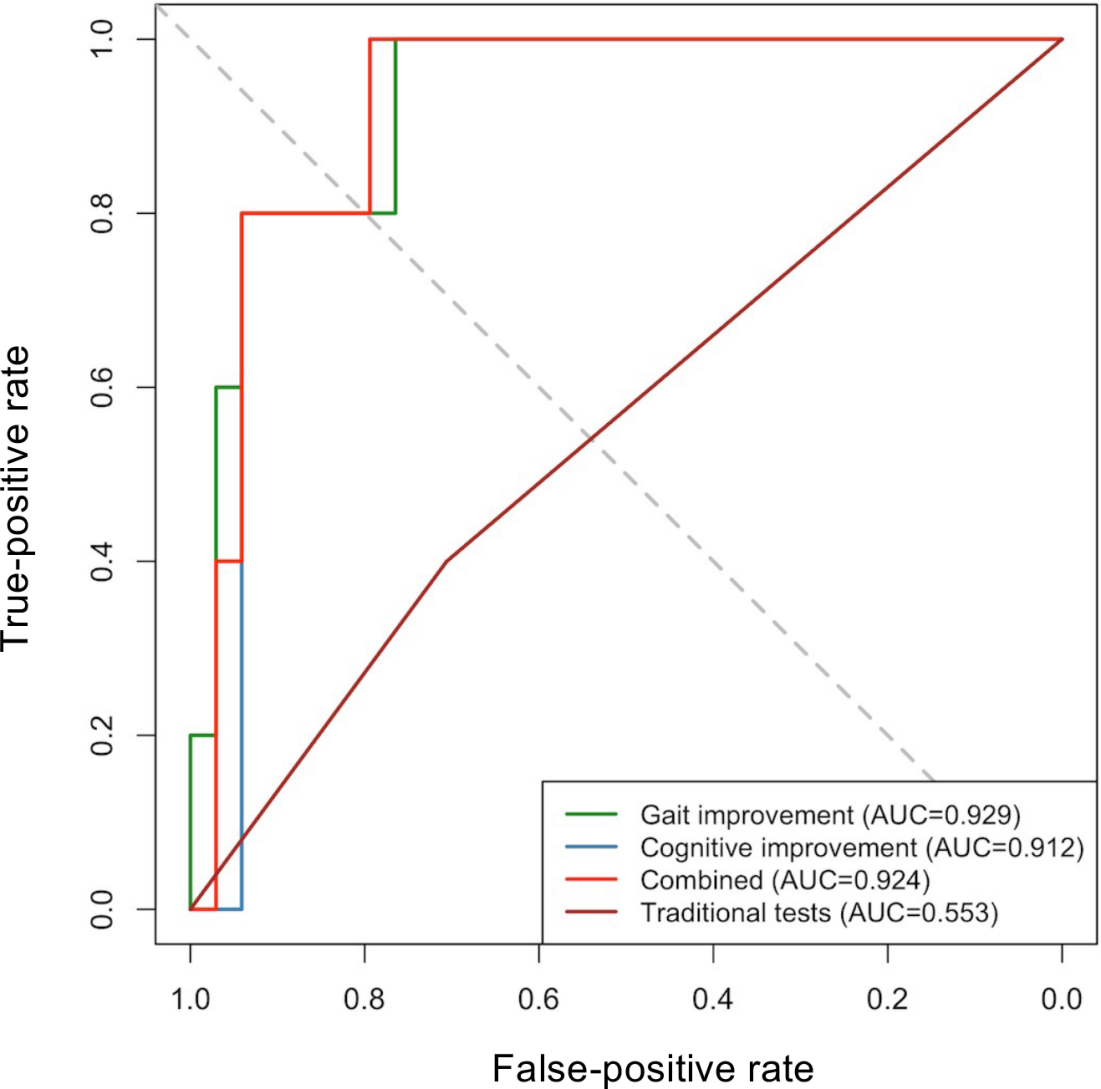
Receiver operating characteristic curve analysis comparing the predictive value of traditional tests, digital neuropsychological and gait tests in differentiating shunt-responders from non-responders. AUC: area under the curve.

As shown in Table 3 and Figure 3, traditional tests in ELD yielded poor diagnostic performance, with an AUC of 0.55 (95% CI 0.30‐0.81), sensitivity of 40% (95% CI 12%‐77%), specificity of 71% (95% CI 54%‐83%), PPV of 17% (95% CI 5%‐45%), and NPV of 89% (95% CI 72%‐96%). In contrast, the combined digital cognitive and gait approach yielded an AUC of 0.92 (95% CI 0.83‐1.00), sensitivity of 100% (95% CI 57%‐100%), specificity of 79% (95% CI 63%‐90%), PPV of 42% (95% CI 19%‐68%), and NPV of 100% (95% CI 88%‐100%). The bootstrap-derived cutoffs showed moderate dispersion, whereas the predictive performances of all digital tests remain relatively stable (lower limits of all AUCs >0.81). The DeLong test demonstrated that combining digital cognitive and gait tests showed better predictive performance of shunt outcome compared to traditional tests (Z=2.43; *P*=.015).

Furthermore, the calibration curve demonstrated that the combined improvement model showed good overall calibration quality (Multimedia Appendix 5, Spiegelhalter *P*=.62), with a low average calibration error of 3.0%, despite slight overconfidence (calibration slope=1.299).

**Table 3.** The predictive efficacy of traditional tests, digital neuropsychological, and gait tests. All CIs were calculated using 2000 bootstrap resamples.

	Cutoff (95% CI)	AUC^a^ (95% CI)	Sensitivity (95% CI)	Specificity (95% CI)	PPV^b^ (95% CI)	NPV^c^ (95% CI)
Traditional tests	0.159 (0.06‐0.29)	0.553 (0.301‐0.805)	0.400 (0.118‐0.769)	0.706 (0.538‐0.832)	0.167 (0.047‐0.448)	0.889 (0.719‐0.961)
Digital tests						
Gait improvement rate after ELD^d^	0.138 (0.10‐0.60)	0.929 (0.830‐1.000)	1.000 (0.566‐1.000)	0.765 (0.600‐0.876)	0.385 (0.177‐0.645)	1.000 (0.871‐1.000)
Cognitive improvement rate after ELD	0.125 (0.08‐0.60)	0.912 (0.812‐1.000)	1.000 (0.566‐1.000)	0.794 (0.632‐0.897)	0.417 (0.193‐0.680)	1.000 (0.875‐1.000)
Combined improvement rate after ELD	0.132 (0.08‐0.62)	0.924 (0.829‐1.000)	1.000 (0.566‐1.000)	0.794 (0.632‐0.897)	0.417 (0.193‐0.680)	1.000 (0.875‐1.000)

a AUC: area under the curve.

b PPV: positive predictive value.

c NPV: negative predictive value.

d ELD: external lumbar drainage.

## Discussion

### Principal Findings

In this study, we investigated the predictive value of digital neuropsychological and gait analyses during ELD for shunt outcome in patients with iNPH. Our findings revealed that while both traditional and digital cognitive and gait assessments improved significantly after 3 days of ELD, the digital tests outperformed traditional testing in terms of predicting shunt outcomes.

Our study found that patients with probable iNPH showed gait improvement after ELD, which was primarily manifested as increased gait speed, longer stride length, higher step height, and shorter turning time. This is consistent with the findings of prior investigations using quantitative gait analysis [47]. Electronic walkways, wearable sensors, and accelerometers are all commonly used for quantitative gait analysis [24,48]. A prospective study using electronic walkways discovered that patients with iNPH had increased stride length and gait speed following a tap test [47]. Another research study has revealed that 72 hours following the tap test, patients with probable iNPH had longer stride lengths, shorter double support times, and faster cadences [49]. A recent study based on 3-dimensional gait analysis indicated that patients with probable iNPH may show improvements in spatiotemporal parameters, such as step length, gait speed, and cadence 24 hours after the tap test, with improvement rates ranging from 4.9% to 10.5% [50]. Besides, a preliminary study using a video-based method qualitatively assessed gait changes after the tap test and found significant improvements in step height [51], whereas earlier studies based on inertial sensors and electronic walkways may not provide precise quantitative assessments of step height. Our vision-based system provided richer spatiotemporal gait parameters than previous methods, without the need for wearable sensors. Therefore, it can provide improvements in step height following ELD, which partially complements the findings of previous studies.

Cognitive impairment is the second most common symptom in patients with iNPH, which may be partly due to mechanical stress to the brain and the existence of Alzheimer disease co-pathologies [25,52]. Frontal lobe dysfunction is thought to be the classic cognitive profile in patients with iNPH [53]. In our study, we observed significant improvements in one-back tests and Stroop color-word tests post-ELD, indicating improvements in executive function, attention, and working memory, all of which are associated with improved frontal lobe functions. Patients with iNPH often show significant improvements in executive subfunction after CSF drainage [54], with prior research showing improvements in verbal fluency, frontal assessment battery, trail-making test, and Stroop color-word tests [54-57]. Therefore, executive subfunction assessment is emphasized in evaluations during CSF drainage to distinguish iNPH from its mimics [54,58]. Previous studies have developed an executive function battery for patients with iNPH based on tests such as TMT-A, Stroop color-word tests, and digit symbol substitution tests [36], and this battery demonstrates a sensitivity of 80% in predicting shunt responders with a specificity of 100%, indicating that evaluating executive function, attention, and reaction time before and after CSF drainage may be crucial in identifying shunt responders [36].

Despite the widespread use of the CSF drainage test for diagnosing and predicting shunt prognosis in patients with iNPH, earlier research indicated that the sensitivity of a single-tap test might be as low as 26%, whereas continuous ELD had a sensitivity of approximately 50% [10,15]. Although the tap test response would be influenced by morphological features and CSF dynamic changes [59], the method used to evaluate the CSF drainage test itself may be crucial for determining its overall sensitivity [2,11]. In the latest Japanese iNPH management guidelines, MMSE, TMWT, and TUG tests were recommended to assess changes in patients’ gait and cognitive function [2]. However, using these methods for assessment may result in very low sensitivity and negative predictive value [11]. A previous large-scale European multicenter study indicated that the negative predictive value of a single lumbar puncture drainage could be as low as 18% [44], with other literature reports generally ranging from 18% to 50% [11]. In our cohort, traditional cognitive and gait tests exhibited a sensitivity of only 40% and a specificity of 70%, which demonstrated that traditional tests may potentially lead to misdiagnosis of shunt responders. Therefore, traditional assessment methods may be insufficient to guide the selection of shunt candidates [2].

In recent years, digital neuropsychological evaluation equipment has emerged that provides randomized test paradigms and automated recording of variables such as reaction time, thereby improving test efficacy [21]. Preliminary research has used digital cognitive and gait evaluation tools for patients with iNPH, establishing computerized neuropsychological tests as reliable techniques for diagnosing cognitive impairments and postoperative cognitive improvements in patients with iNPH [22]. In contrast, a recent meta-analysis suggested that quantitative gait analysis can detect gait improvements in patients with iNPH at baseline, after the tap test, and postshunt [60]. These sensor-based technologies and digital systems possess high scalability, offering continuous and high-precision monitoring of cognitive and motor functions [61]. Our results further emphasized the predictive value of these digital assessment tools during ELD for patients with iNPH, which also showed better performance in predicting shunt responses. Therefore, the use of digital evaluation tools may aid in the selection of potential shunt candidates. Integrating these digital data into telehealth platforms would enable remote and continuous health care for patients with geographic barriers, reduce hospital visits, and enhance patient outcomes [61]. However, the results of our study are still preliminary; research gaps for digital assessments still exist in modest sample size, clinical standardization, and multicenter validation. Future research should explore more data-driven weighting schemes and conduct external validation as more data become available.

### Strengths and Limitations

The main advantages of our study are the prospective cohort study design, which collected detailed clinical data, and comprehensive neuropsychological and gait assessment data from the patients. Additionally, we are the first to combine computerized neuropsychological assessment and 3-dimensional gait analysis to evaluate symptom changes during ELD in patients with iNPH. The limitations of the study are as follows: (1) Nearly half of the patients with positive ELD responses refused shunt surgery, and the relatively small sample size of shunted patients may constrain the statistical power, despite our use of Firth penalized regression models. Therefore, the relatively high AUC and sensitivity values should be interpreted with caution. Future multi-center studies with larger samples are needed to validate our preliminary findings. (2) Cut-off values were calculated statistically based on the Youden index. Given the relatively small sample size, these data-derived thresholds should be interpreted as preliminary estimations that require external validation in future studies with larger cohorts. (3) The high rate of shunt refusal may inevitably introduce selection bias, although the baseline characteristics between patients who accepted and refused shunts were largely comparable, except for DESH score (Table 1). (4) Several patients were excluded because of severe illness; therefore, these results may be primarily applicable to patients who have iNPH with mild-to-moderate symptoms. (5) In this study, quantitative assessments were only completed on the third day after ELD. Although previous studies indicate that a 3-day continuous ELD is sufficient to improve cognitive and gait function in patients with iNPH [13,49], a recent longitudinal study suggests that patients with iNPH would exhibit delayed response up to 2 weeks after ELD [27]. Therefore, further research is needed to determine the best evaluation time point. (6) Due to the relatively short follow-up interval, the long-term predictive value of these digital tests for shunt response was unclear, which may be affected by various prognostic factors [62]. (7) Although our findings on digital assessments are promising, the generalizability across different health systems and the cost-effectiveness of these digital assessments are underexplored.

### Conclusions

Digital neuropsychological and motor assessments could identify gait and cognitive improvements after continuous ELD. Moreover, the digital tests showed better predictive performance for shunt outcome compared to traditional tests in our pilot study. Further validation of our findings in a multicenter setting is required to establish the optimal cutoff values for these digital evaluation tests.

## Supplementary material

10.2196/78399 Multimedia Appendix 1 Participant flow.

10.2196/78399 Multimedia Appendix 2 Proportion of symptom combinations in patients with idiopathic normal pressure hydrocephalus.

10.2196/78399 Multimedia Appendix 3 Changes in cognitive and gait function after 3-day external lumbar drainage.

10.2196/78399 Multimedia Appendix 4 Firth logistic regression models of digital evaluation methods for differentiating shunt responders from nonresponders.

10.2196/78399 Multimedia Appendix 5 Calibration curve of the combined improvement model.

10.2196/78399 Checklist 1 STROBE checklist.

## Abbreviations

AUC: area under the curve
CSF: cerebrospinal fluid
ELD: external lumbar drainage
iNPH: idiopathic normal pressure hydrocephalus
iNPHGS: idiopathic normal pressure hydrocephalus grading scale
MMSE: Mini-Mental State Examination
mRS: modified Rankin Scale
OR: odds ratio
SCWT: Stroop color-word test
STROBE: Strengthening the Reporting of Observational Studies in Epidemiology
TMT: trail-making test
TMWT: 10-m walking test
TUG: timed up and go
