# Supplementary figures and images for: Predictive Value of Digital Neuropsychological and Gait Assessments on Shunt Outcome in Patients With Idiopathic Normal Pressure Hydrocephalus: Prospective Cohort Study
Source: J Med Internet Res. 2025 Nov 25;27:e78399. doi: 10.2196/78399 (PMC12646562; doi:10.2196/78399)

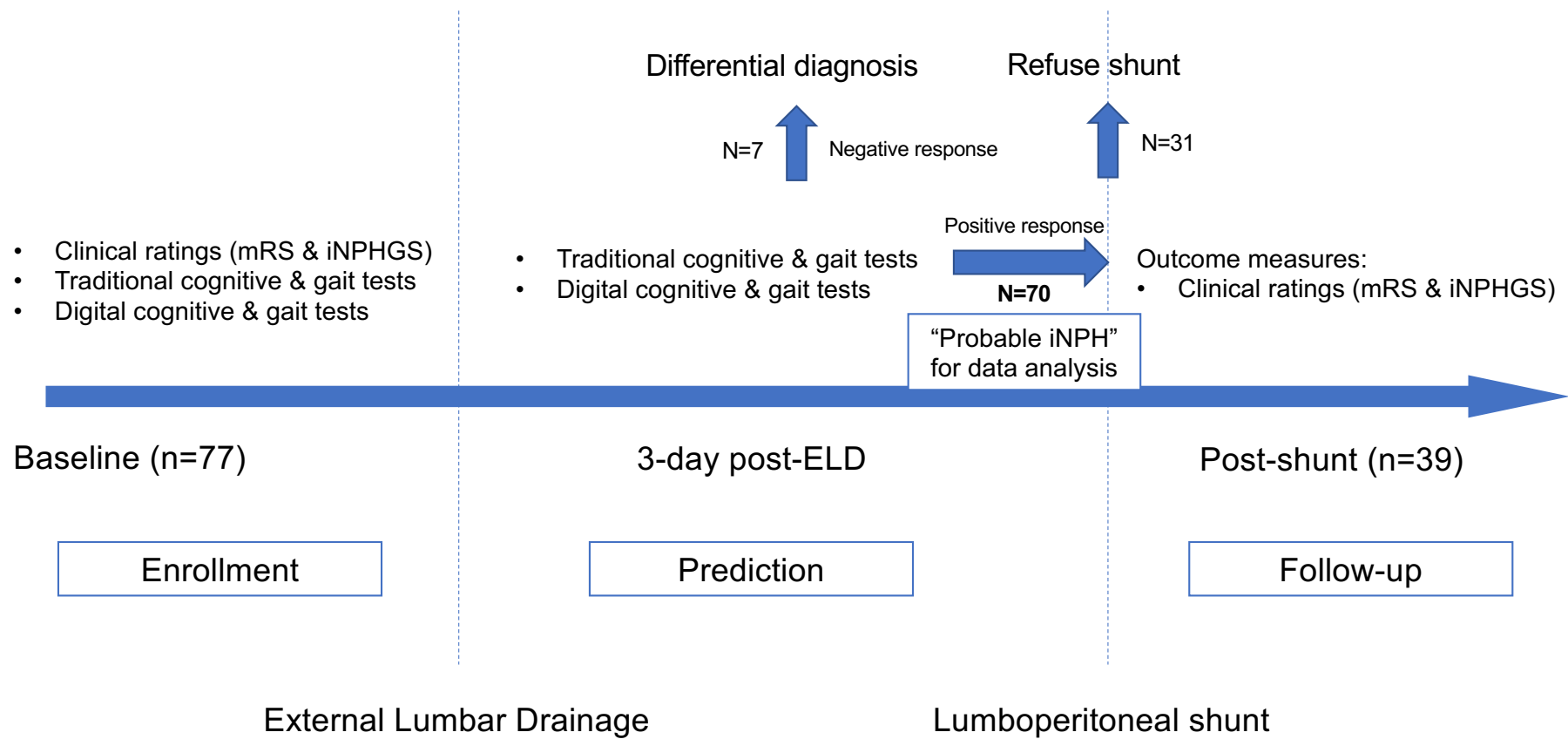

Supplement: Multimedia Appendix 1 [file jmir-v27-e78399-s001.pdf]

**Supplementary Figure 2. Proportion of symptom combinations in patients with iNPH.**

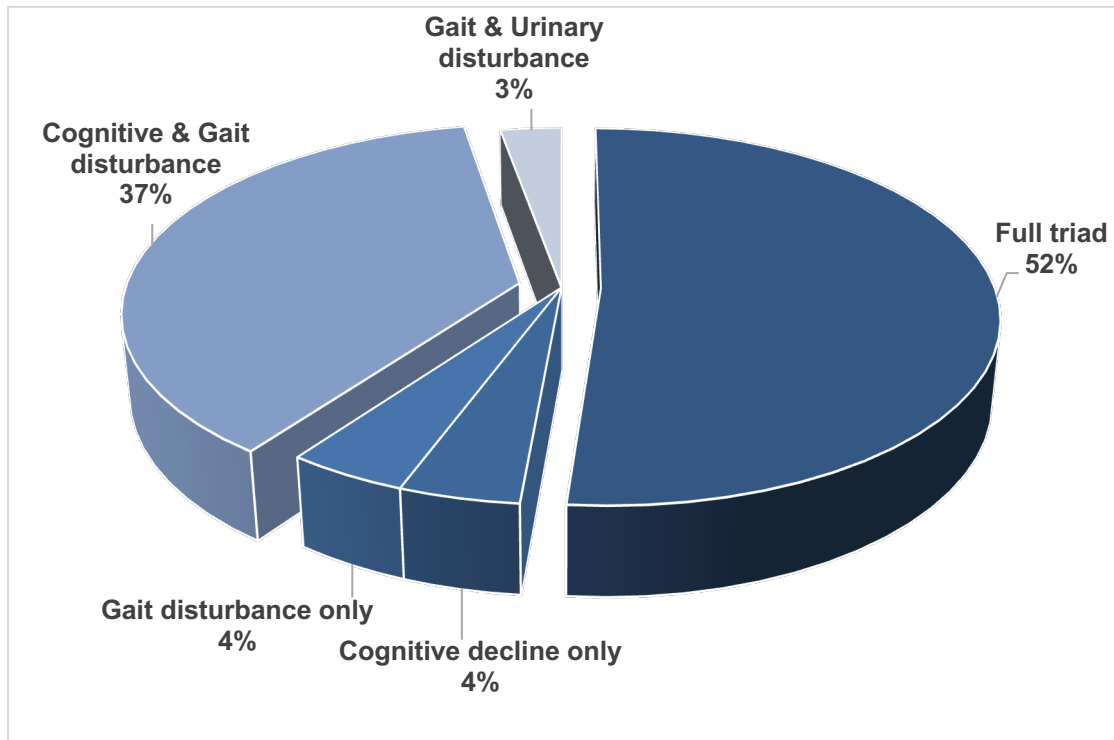

Supplement: Multimedia Appendix 2 [file jmir-v27-e78399-s002.pdf]

**Supplementary Figure 3. Calibration curve of the combined improvement model.**

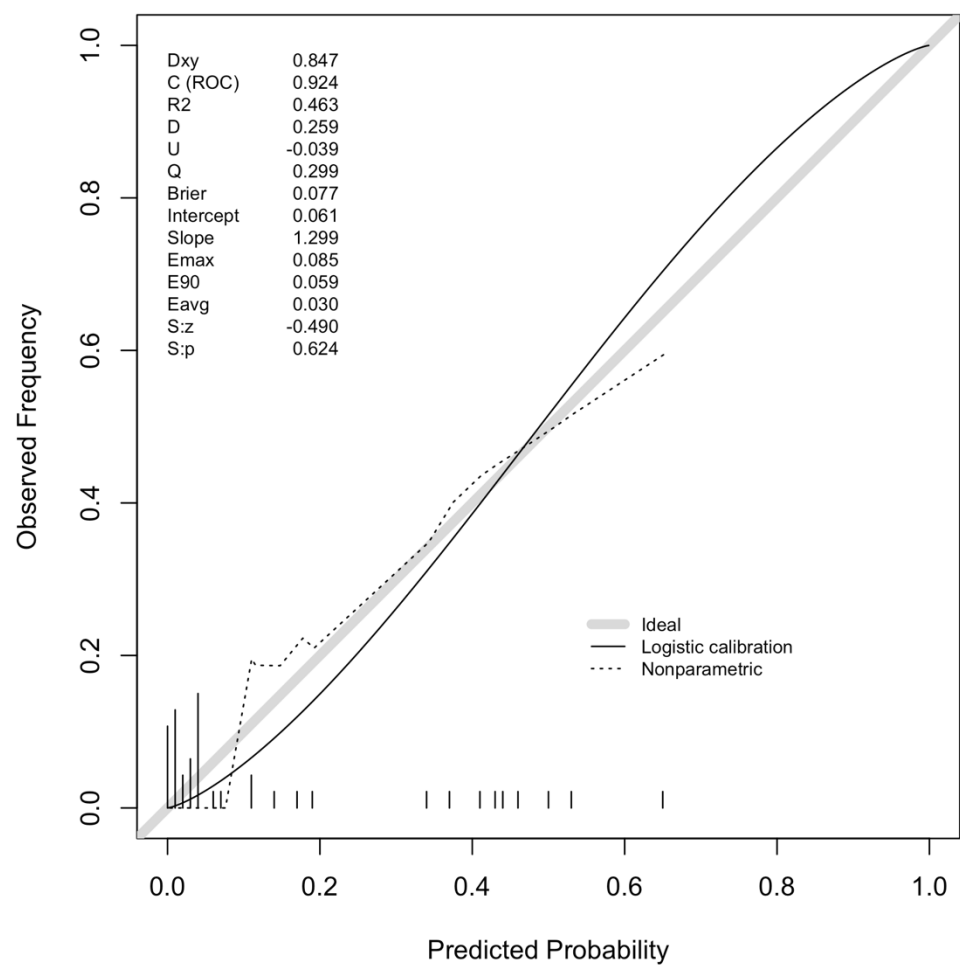

Supplement: Multimedia Appendix 5 [file jmir-v27-e78399-s005.pdf]
