# Supplementary material for: Predictive Value of Digital Neuropsychological and Gait Assessments on Shunt Outcome in Patients With Idiopathic Normal Pressure Hydrocephalus: Prospective Cohort Study
Source: J Med Internet Res. 2025 Nov 25;27:e78399. doi: 10.2196/78399 (PMC12646562; doi:10.2196/78399)
Supplement: Multimedia Appendix 3 [file jmir-v27-e78399-s003.pdf]

Supplementary Table 1. Changes of cognitive and gait function after 3-day ELD.

Variables	Baseline (n=70)	3-day post-ELD (n=70)	p	Methods
**Traditional tests**				
MMSE[b], scores	21.0[14.0, 25.0]	23.0[17.0, 27.0]	**<0.001***	Wilcoxon
5m-TUG[b], s	18.0[15.0, 27.0]	18.0[13.0, 24.0]	**0.004***	Wilcoxon
TMWT-time[b], s	15.0[12.0, 22.0]	14.0[11.0, 21.0]	**0.016***	Wilcoxon
TMWT[b], steps	26.0[21.0, 38.0]	24.0[18.0, 32.0]	**<0.001***	Wilcoxon
**Digital tests**				
Grammatical reasoning[b], scores	1.0[0.0, 3.0]	1.0[-1.0, 6.0]	0.788	Wilcoxon
One-back test[b], scores	5.0[2.0, 9.0]	8.0[3.0, 12.0]	**0.01***	Wilcoxon
Trail-making test[b], s	140.0[103.0, 150.0]	134.0[98.0, 150.0]	0.42	Wilcoxon
SCWT[b], scores	36.0[14.0, 45.0]	40.0[28.0, 48.0]	**0.009***	Wilcoxon
SCWT-time[b], s	150.0[116.6, 186.7]	147.4[127.5, 178.2]	0.9	Wilcoxon
Cognitive z-score[a], scores	-2.35±1.19	-2.06±1.30	**0.025***	related t-test
Step width[b], m	0.16[0.14, 0.17]	0.16[0.14, 0.17]	0.488	Wilcoxon
Stride length[b], m	1.35[0.85, 1.72]	1.38[1.07, 1.79]	**0.01***	Wilcoxon
Step height[a], m	0.079±0.025	0.085±0.023	**0.048***	related t-test
Gait velocity[a], m/s	0.676±0.221	0.721±0.218	**0.019***	related t-test
Turning time[b], s	2.22[1.57, 3.40]	1.78[1.38, 2.76]	**<0.001***	Wilcoxon

Notes: MMSE, mini-mental state examination; SCWT, Stroop color-word test; TUG, timed-up and go test; TMWT, ten-meter walking test; ELD, external lumbar drainage.

*, Statistically significant.

[a] Normally distributed data are expressed as the mean ± standard deviation. Paired samples t-test was used for group comparisons.

[b] Non-normally distributed data are reported as medians (interquartile range). Wilcoxon signed-rank test was used for group comparisons.
