# Supplementary material for: Predictive Value of Digital Neuropsychological and Gait Assessments on Shunt Outcome in Patients With Idiopathic Normal Pressure Hydrocephalus: Prospective Cohort Study
Source: J Med Internet Res. 2025 Nov 25;27:e78399. doi: 10.2196/78399 (PMC12646562; doi:10.2196/78399)
Supplement: Multimedia Appendix 4 [file jmir-v27-e78399-s004.pdf]

Supplementary Table 2. Firth's logistic regression models of digital evaluation methods for differentiating shunt responders from non-responders.

Variables	Univariate				Multivariate			
	β	S.E	P	OR (95%CI)	β	S.E	P	OR (95%CI)
Gait improvement, %	-0.07	0.04	0.08	0.93 (0.854 ~ 1.01)	-0.10	0.05	**0.03***	0.90 (0.78 ~ 0.99)
Cognitive improvement, %	-0.01	0.01	0.122	0.99 (0.98 ~ 1.00)	-0.01	0.01	0.065	0.99 (0.98 ~ 1.00)
Combined improvement, %	-0.02	0.01	0.072	0.98 (0.96 ~ 1.00)	-0.02	0.01	**0.032***	0.98 (0.95 ~ 1.00)

Notes: S.E, standard error; OR, odds ratio; CI, confidence interval; *, statistically significant. All P-values are based on permutation tests with 5,000 iterations.
